# Optimization an Optimal Artificial Diet for the Predatory Bug *Orius sauteri* (Hemiptera: Anthocoridae)

**DOI:** 10.1371/journal.pone.0061129

**Published:** 2013-04-05

**Authors:** Xiao-Ling Tan, Su Wang, Fan Zhang

**Affiliations:** 1 Institute of Plant & Environment Protection, Beijing Academy of Agriculture & Forestry Sciences, Beijing, China; 2 Key Laboratory of Applied Entomology, Northwest A&F University, Yangling, Shannxi, China; CNRS, Université de Bourgogne, France

## Abstract

**Background:**

The flower bug *Orius sauteri* is an important polyphagous predator that is widely used for the biological control of mites and aphids. However, the optimal conditions for mass rearing of this insect are still unclear, thus limiting its application.

**Methodology:**

In this study, we investigated the optimal ingredients of an artificial diet for raising *O. sauteri* using a microencapsulation technique. The ingredients included egg yolk (vitellus), whole-pupa homogenate of the Tussah silk moth (*Antheraea paphia*), honey, sucrose, rapeseed (*Brassica napus*) pollen and sinkaline. We tested 25 combinations of the above ingredients using an orthogonal experimental design. Using statistical analysis, we confirmed the main effect factors amongst the components, and selected five optimal combinations based on different biological and physiological characters.

**Principal Findings:**

The results showed that, although different artificial diet formats significantly influenced the development and reproductive ability of *O. sauteri*, the complete development of *O. sauteri* to sexual maturity could only be achieved by optimizing the artificial diet according to specific biological characters. In general, pupae of *A. paphia* had more influence on *O sauteri* development than did artificial components. The results of a follow-up test of locomotory and respiratory capacity indicated that respiratory quotient, metabolic rate and average creeping speed were all influenced by different diets. Furthermore, the field evaluations of mating preference, predatory consumption and population dispersion also demonstrated the benefits that could be provided by optimal artificial diets.

**Conclusions:**

A microencapsulated artificial diet overcame many of the difficulties highlighted by previous studies on the mass rearing of *O. sauteri*. Optimization of the microencapsulated artificial diet directly increased the biological and physiological characters investigated. Successive physiological tests and field investigations were used to evaluate the outcome of different artificial diet combinations on the quality of the reared *O. sauteri*.

## Introduction

The use of the predacious flower bug, *Orius sauteri* (Heteroptera: Anthocoridae) as a method of biological control in greenhouses was first demonstrated over 10 years ago [1]. Several hemipteran, thysanopteran and lepidopteran pests can be efficiently controlled by *O. sauteri* [2]–[8]. This efficient predator is indigenous to Asia and its biology, physiology and ecology have been well studied [8]–[12]. However, a key limitation to the extensive application of *O. sauteri* as a means of biological control, particularly of pests of food crops, is the lack of understanding of the requirements for rearing stable laboratory populations of *O. sauteri*, which is required for the mass production of these insects for inundative or inoculative release [13]. A better understanding of the regulation of rearing conditions, preferential oviposition substrates and optimal diet combinations is vital for the successful artificial massive rearing of *O. sauteri* [14]–[17].

The use of artificial diets to rear biological control arthropod agents has been studied for over 30 years [18]. As with other predacious *Orius* species (e.g. *O. tristicolor*, *O. laevigatus* and *O. albidipennis*), introducing natural prey as the primary diet was the initial method used for artificial rearing of *O. sauteri* [19]–[23]. However, a natural prey diet was unable to support the *O. sauteri* population throughout the year because of environmental condition limitations and the need to rear prey. Therefore, successful continuous mass rearing of *O. sauteri*, particularly at a commercial level, required the development of alternative artificial diets, which were initially based on the artificial rearing of other *Orius* species. Previous studies showed that water-soluble botanic substances, such as flower pollen, had a prominent role in the artificial diet in terms of the growth of *Orius* species [20], [21], [24]. Similarly, artificial diets used in the mass rearing of *Orius* that included animal splanchnic tissue, insect cell lines or meridic egg yolk protein were known to be metabolized for nutrition and energy by the insects [25]–[28]. In addition, chemical substances, such as saccharides or choline, were included as necessary components of the diet [29]. Despite these studies, an optimal artificial diet is still lacking for successful rearing of *O. sauteri*.

In addition to the components of the diet, its state (liquid, solid, etc.) can also influence rearing success, given that *Orius* species have sucking mouth parts only. Although a promoted liquid diet could be easily ingested by sucking in *Orius similis*, it led to high insect mortality because the liquid feed was sticky [30]. However, a solid artificial diet is not suitable for insects that feed using sucking mouthparts. In addition, a solid diet is also unsuitable for rearing *Orius* species because it can easily become contaminated and dried out [31], [32]. Moreover, the liquid and solid forms of previously attempted artificial diet did not meet the commercial requirements in terms of acting as a nutrition reserve during storage and transportation of biological control agents. To overcome these issues, microencapsulation was introduced, which is an advanced packaging technique in widespread use for packaging microbial agents and chemical or food products in the form of microcapsules to promote the quality of artificial diets [33], [34]. Although the development of an optimal artificial diet to maintain a constant supply of insects has been under continual study, manufacturing such a diet for insects with sucking mouthparts has been more difficult [35]. Earlier work from our laboratory confirmed the optimal proportions of the basic ingredients for a microencapsulated artificial diet based on its effects on the development and reproductive capacity of *O. sauteri* [36]. Based on these results, we developed an integrated artificial diet recipe using microencapsulation for the mass rearing of *O. sauteri* for commercial biological control application.

The aim of the current study was to determine the optimal integrated microencapsulated artificial diet for *O. sauteri* based on the developmental and reproductive status of the insect, as determined using orthogonal tests. We also investigated the effects of the different diets on the locomotory and respiratory performance, mating preference, predatory ability and population dispersion of *O. sauteri* compared with individuals fed on insect prey.

## Materials and Methods

### Stock colony

Adult *O. sauteri* adults were collected from an organic apple orchard (40°14′N, 116°13′E) in Changping County, Beijing, China during July to September, 2009. A total of 1024 adults (551 males and 473 females) were collected and reared in the laboratory at the Institute of Plant & Environment Protection, Beijing Academy of Agriculture & Forestry Sciences, Beijing, China, under the following environmental conditions: 25±1°C, 16L∶8D light∶dark period, and 70% relative humidity (RH). The environmental illumination intensity was less than 4000 lx, as determined by a set of indoor automatic environment regulation devices for sample rearing. Healthy *Orius sauteri* were selected from the field collection and 25 males and females were reared in a custom-made cage (20.0×25.0×40.0 cm, aluminum alloy+plastic optical fiber net). The reproduction of these experimental populations depended on a daily supply of *Tetranychus cinnabarinus* mites as prey and fresh white kidney bean *Phaseolus vulgaris* seedlings as an ovipositioning substrate. Newly hatched nymphs were selected for use in the study.

### Optimization of artificial diets for *O. sauteri* rearing

#### Artificial diet ingredients and orthogonal collocation

An orthogonal L_25_^(56)^ test design was used to investigate the optimal artificial recipes according to the developmental and reproductive performances of *O. sauteri*. As shown in Table 1, the optimization experiments were performed using six ingredients and five concentration gradients, as follows: egg yolk (0, 10, 20, 30, 40 g), tussah pupae lyophilized powder (10, 20, 30, 40, 50 g), sucrose (as a feeding stimulant [37], 0, 5, 10, 15, 20 g), honey (, 0, 5, 10, 15, 20 g), rape pollen (0, 5, 10, 15, 20 g) and sinkaline (0, 5, 10, 15, 20 g). The range of the concentration gradient of each ingredient was measured in preliminary tests. In total, 25 types of artificial diet were investigated.

**Table 1:** The composition of each orthogonal experiment treatment.

Recipes	Composition of artificial diet (g)					
	Egg yolk (A)	Tussah pupa (B)	Sucrose (C)	Honey (D)	Rape pollen (E)	Sinkaline (F)
1	0	10	0	0	0	0
2	0	20	5	5	5	5
3	0	30	10	10	10	10
4	0	40	15	15	15	15
5	0	50	20	20	20	20
6	10	10	5	10	15	20
7	10	20	10	15	20	0
8	10	30	15	20	0	5
9	10	40	20	0	5	10
10	10	50	0	5	10	15
11	20	10	10	20	5	15
12	20	20	15	0	10	20
13	20	30	20	5	15	0
14	20	40	0	10	20	5
15	20	50	5	15	0	10
16	30	10	15	5	20	10
17	30	20	20	10	0	15
18	30	30	0	15	5	20
19	30	40	5	20	10	0
20	30	50	10	0	15	5
21	40	10	20	15	10	5
22	40	20	0	20	15	10
23	40	30	5	0	20	15
24	40	40	10	5	0	20
25	40	50	15	10	5	0

#### Production of artificial diet microcapsules

The raw materials [egg yolk, whole Tussah pupae homogenate, sucrose, honey, rapeseed pollen and sinkaline (choline chloride)] were dissolved in distilled water in a clean 300-mL glass beaker using the concentrations shown in Table 1; the final constant volume was filled to 500 mL in a volumetric flask. The mixed artificial diet solutions from No. 1 to No. 25 were used as the core material in the microencapsulation process.

**Figure 1:**
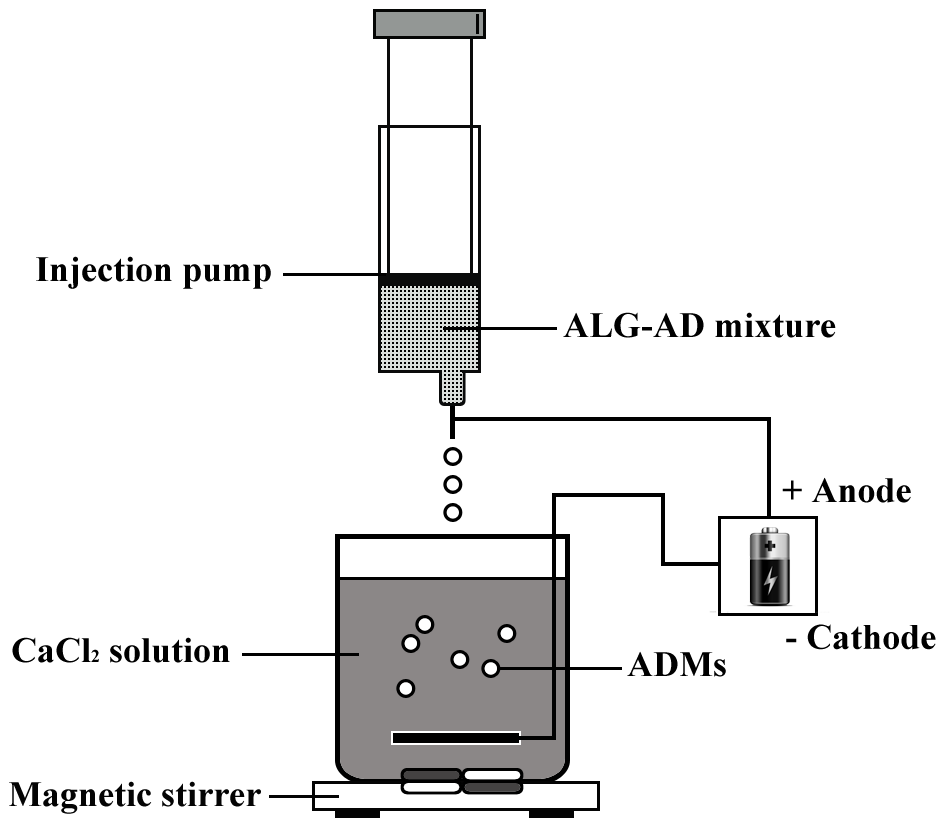
The device used in the microencapsulation process by means of the complex coacervation method.

A general complex coacervation method [38] was used to make the artificial diet microcapsules (ADMs). In a previous study, we had determined the optimal basic chemical materials for artificial diet microencapsulation for feeding *O. sauteri* to be as follows: sodium alginate 2%, chitosan 0.6% and a 13∶1 ratio of core material to wall-forming material [35]. Production of the ADMs involved eight steps: (i) raw material treatment; (ii) sterilization; (iii) proportioning; (iv) emulsification; (v) granulation; (vi) rinsing; (vii) filtration; and (viii) packaging. In the first instance, we weighed quantificational calcium chloride (analytically pure, SCRC) using an electronic capacity balance (Figure 1). The ADMs were made as follows: a 2% mass concentration calcium chloride solution was prepared using distilled water. A prepared artificial diet solution and quantificational sodium alginate (at the previously mentioned concentration) were mixed using a magnetic stirrer for 5 min at 3000 rpm to ensure uniform mixing. The mixture was then added to a Top-5300 model medical micro-injection pump (Top Corporation, Japan) (inside chamber cubage: 50 ml; range of flow setting: 0.1–1500.00 ml/h). We adjusted the pinhead of the injection pump to almost 5.0 cm to the surface of the calcium chloride solution. After turning on the power, the injection pump dropped the mixture into the calcium chloride solution under 5 bars. The dropping speed could be controlled by adjusting the pressure of the compressed air. The ADMs prepared were maintained in calcium chloride solution for at least 5 min to establish their stability, and were then moved to a quantificational chitosan solution and shocked regularly for 13 min. This resulted in calcium alginate-chitosan-sodium alginate colloid particles that were kept in 0.15% sodium alginate solution for 30 min and then put into a 0.055 mol/L sodium citrate solution for 10 min. After being rinsed in physiological saline, the microcapsules were also gently washed with distilled water five times. Finally, the ADMs were conserved in airproof plastic bags and stored at 5±1°C until required.

We produced 25 co-allocated groups of ADMs based on the orthogonal setup detailed in Table 1. In total, 1000 microcapsules were prepared for each 25 ADM group.

#### Rearing investigation of ADMs according to developmental and reproductive performance

To confirm the optimal ingredients of the artificial diet, the ADMs were offered to new nymphs under the controlled environment conditions described above in a MLR-351H model manual environmental chamber (Sanyo, Japan). Each nymph was removed to a clean Petri dish (D = 4.5 cm) with four ADM and one fresh *P. vulgaris* seedling for 12 h. The Petri dish was covered with parafilm with several holes in it to aid ventilation. We then recorded the total nymphal development duration and survival ratio once they had pupated to adults. We then introduced one virgin male (or female) to the Petri dish and recorded the oviposition duration and level of female fertility (net hatched 1^st^ instar nymph of offsprings) until the female was dead. *Orius sauteri* fed only on *T. cinnabarinus* under the same environmental conditions were used as the control.

For each orthogonal group, five replications, each with ten nymphs, were performed.

### Bioassay of *O. sauteri* fed on optimal ADMS

Based on the data from the above experiment, we produced ADMs containing the four recipes that had resulted in optimal performance of selected biological traits: total nymphal development duration (ADM-D), survival ratio (ADM-S), oviposition duration (ADM-O) and female fertility (ADM-F), and which were then offered to adult female *O. sauteri*. *Orius sauteri* female adults reared with *T. cinnabarinus* were used as controls. Except for the predation experiment, the bioassays were conducted under regulated environmental conditions as mentioned above.

#### Respiratory and locomotory performance

The respiratory rate of the insects was determined using a respiratory measurement device (Sable System Co. Ltd) that comprised Testo-535 model CO_2_ infrared analyzers (Testo, Deutschland), an InPro-6000G model gas O_2_ sensor (Mettler-Toled, Switzerland), an air pump, a flow meter, a CO_2_ purification tube, a drying tube, a glass sample chamber (2 cm ID×10 cm), a LabPro data collector (Vernier, USA) and a TMP-112 model temperature sensor (Texas Instrument, USA). We adjusted the air pump to keep the air flow at 0.1 L/min. The CO_2_ infrared analyzers and O_2_ sensor were then switched on to determine the initial CO_2_ and O_2_ concentrations. When these levels were steady, we placed a newly emerged *O. sauteri* adult female that had been fed with the ADM consistently into the sample chamber for 30 min, during which time we recorded the temperature, CO_2_ and O_2_ concentration at the end of the 30-min period. All experiments were conducted at 25±1°C.

We calculated the respiratory quotient (RQ) and respiratory rate (RR) using the following formulae:
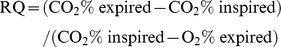


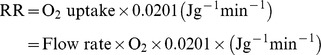



In addition, we used newly emerged *O. sauteri* female adults fed on *T. cinnabarinus* as controls. Each experimental treatment and control group was replicated five times with ten adult females.

We utilized LC-100 Tracksphere (Synthetic, Germany) to compare the locomotory capacity of *O. sauteri* adults reared on optimal ADMs (as mentioned above) or on *T. cinnabarinus* (controls). After adjusting the focus, we placed a newly emerged *O. sauteri* adult female on the top of the monitoring sphere. The sphere adjusted the moving speed automatically and ensured that the sample was in a relative rest position. We then observed the locomotory status of each insect over 5 min and recorded the average creep speed.

Each optimal ADM treatment and control group was replicated five times, with ten adults each replicate.

#### Mating preference of wild O. sauteri

In total, 361 adult females and 417 adult males of *O. sauteri* were collected from maize fields at the Institute of Botany, Chinese Academy of Sciences (Haidian, Beijing) in July, 2010 as a wild experimental population. Fifty pairs of healthy adults were selected for mating preference experiments. Four copulatory groups were established: ADM-reared male (A♂)+wild female (W♀), wild male (W♂)+ADM-reared female (A♀), ADM-reared male (A♂)+ADM-reared female (A♀) and wild male (W♂)+wild female (W♀). A pair of *O. sauteri* adults was then placed into a experimental box (0.15×0.20×0.18 cm, made by aluminum alloy as frames +100 mess fabric net as walls). The time of placement was recorded as the start of the mate selection, and the end of the mate selection process was recorded as the point at which the sexual organs of the male and female came into contact and did not separate for a period of 5 min. This ‘time to copula’ was used in the comparison of mating preference differences among all four copulatory groups.

The observations were replicated 25 times for each copulatory group.

#### Predatory ability of ADM-fed O. sauteri

A 1.0×1.0 m test arena with eight kidney bean plants (each 25–30 cm in height, with five leaves each) covered by a cubic cage (1.0×1.0×1.0 m, made by aluminum alloy as frames +100 mess fabric net as walls) was confirmed as a suitable piece of apparatus in glass greenhouses at the agricultural research station of BAAFS (Haidian, Beijing). Ten newly emerged adult female *O. sauteri* were selected from each optimal ADM-reared experimental population. The females were placed in a plastic petri dish and maintained for 12 h without food. A total of 300 *T. cinnabarinus* mites and the five selected *O. sauteri* were then released into the cage simultaneously. We monitored the predation rate of *O. sauteri* and counted the residual number of *T. cinnabarinus* every 8 h for a total of 48 h. In addition, we repeated the experiment with 300 western flower *Frankliniella occidentalis* 4^th^ instar larvae as the target prey.

Each optimal ADM-reared treatment and control group was replicated 15 times for both *T. cinnabarinus* and *F. occidentalis* experiments.

#### Population dispersal in greenhouses

**Figure 2:**
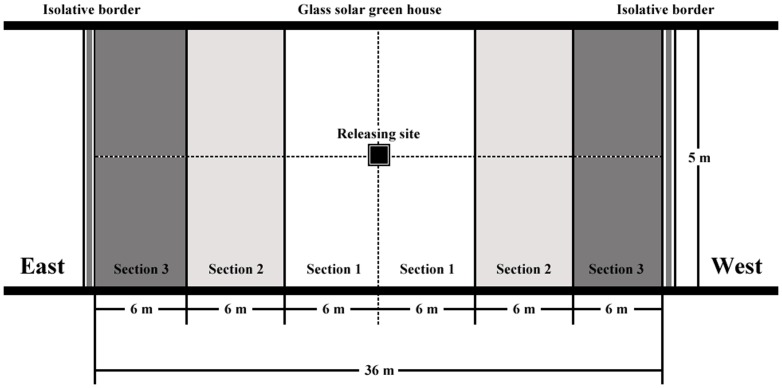
Observational sections in the field test of dispersal of populations of O. sauteri fed optimal ADMs.

In total, 200 optimal ADM-D diet-fed newly emerged *O. sauteri* female adults were maintained in a transparent plastic box (15×20×20 cm) with a daily supply of abundant ADMs. The comparison of *O. sauteri* dispersal rates was conducted in a glass solar greenhouse from 2 to 8 September, 2012, with 2000 tomato *Lycopersicon esculentum* var. *cerasiforme* plants in an experimental area of 180 m^2^ (36×5 m). The experimental area was divided into three sections as shown in Figure 2. The prey, leaf mites *T. cinnabarinus*, were added to the plants at a density of >15 mites per 30 cm^2^ leaf area in each experimental section. The plastic box was placed at the release site and the cover removed. Ten tomato plants were selected random on which to check the number of *O. sauteri* in each experimental section at 24 h, 48, and 72 h. We also observed the population dispersal rate of *O. sauteri* fed on the other three optimal ADMs, and on *T. cinnabarinus* (control group). Each optimal ADM-reared treatment and control group was replicated ten times.

### Statistical analysis

SPSS® 16.0 software was used for the statistical analysis. We calculated the average value of each set of observed data as the mean±SE. The differences in each biological and physiological character were compared using a one-way analysis of variance (ANOVA) among all experimental recipe groups. We analyzed the differences in the duration of total nymphal development, survival ratio and other reproductive factors in the orthogonal experiments to confirm the optimal ADM ingredients and their related proportions using variance and range analysis. Furthermore, the one-way ANOVA was run with the different ingredients comprising the ADMs as independent factors, to compare the differences in respiratory quotient, locomotory capacity, mating preference (time to copula), predatory capacity (residual number of prey in different time checkpoints) and population dispersion (amount of *O. sauteri* in different sections of distance in different temporal checkpoints).

## Results

### Investigation of optimal ADM recipes according to biological characters

#### Total nymphal development duration and survival ratio

In total, 25 groups of ADMs were produced to feed *O. sauteri* and the development duration from newly hatched first instar nymphs to adult eclosion was recorded (Table 2). The total nymphal development duration showed significant differences among all ADM recipes (*F_nymph_* = 89.450, d.f. = 24,1078, *P _total nymph_*<0.001). With the exception of recipe No. 24, all the artificial diets increased the total developmental duration compared with the control.

**Table 2:** Developmental and reproductive characteristics of *O. sauteri* fed on the different ADMs.

Recipes	Total nymphal duration	Survival ratio	Oviposition duration	Female fertility	Recipe	Total nymphal duration	Survival ratio	Oviposition duration	Female fertility
1	23.03±0.83	14.0±4.9	4.35±0.77	2.8±1.8	14	21.07±1.25	35.2±4.0	4.35±0.77	4.0±0.2
2	19.29±0.62	49.0±11.7	4.89±0.93	6.3±1.1	15	19.60±0.62	72.4±4.2	4.89±0.93	21.8±1.0
3	21.22±1.10	83.8±4.4	5.84±0.29	15.5±2.3	16	21.48±0.61	87.5±7.2	5.84±0.29	24.4±2.1
4	20.63±0.97	84.6±10.5	5.16±1.02	9.9±1.1	17	17.44±0.51	74.4±5.2	5.16±1.02	12.9±1.5
5	20.09±0.59	50.0±8.5	3.81±1.36	5.2±0.7	18	17.61±0.75	83.8±3.5	3.81±1.36	11.8±1.4
6	20.66±0.92	24.4±6.4	3.21±0.53	4.8±0.6	19	18.60±1.05	41.4±8.8	3.21±0.53	29.7±2.7
7	19.58±0.85	75.6±10.5	7.31±0.43	20.2±2.8	20	19.31±0.60	30.4±6.5	7.31±0.43	9.9±0.8
8	22.85±0.61	41.4±6.9	3.15±0.51	14.5±2.2	21	19.84±0.54	72.6±4.3	3.15±0.51	34.8±1.6
9	17.24±0.99	48.4±14.4	3.45±0.76	10.1±1.5	22	20.57±0.69	26.2±3.7	3.45±0.76	16.8±2.1
10	18.13±0.55	79.6±8.7	6.38±0.36	29.5±1.1	23	20.88±0.38	24.6±6.2	6.38±0.36	12.1±1.9
11	22.96±1.30	36.2±4.8	3.37±0.76	13.7±1.7	24	15.16±0.33	82.4±7.1	3.37±0.76	42.2±1.9
12	21.72±0.49	75.4±5.2	4.32±0.42	6.3±1.8	25	21.95±0.41	80.2±6.8	4.32±0.42	36.2±1.7
13	24.27±0.57	27.8±6.6	3.14±0.32	18.1±2.0	Control	15.76±0.58	91.3±3.5	3.14±0.32	60.1±2.5

The orthogonal test results indicated that the Tussah pupa was the ADM ingredient that impacted development the most. Based on these results, the order of ingredients in terms of their impact on development duration was as follows: Tussah pupa>egg yolk>sinkaline>sucrose>honey>rapeseed pollen. Intensive range analysis showed that the experimental group with the following ingredients was the most efficient for feeding: egg yolk 30 g, Tussah pupa 40 g, sucrose 10 g, honey 15 g, sinkaline 20 g and no rapeseed pollen. We also recorded the total nymphal development duration of *O. sauteri* after feeding with the optimal ADM, and this was found to be 14.80 d.

Not all nymphs fed ADMs developed into adults. The survival of nymphs across all ADM diets was lower than in the control group; in addition, different ingredients significantly influenced the mortality of *O. sauteri* (Table 2; *F* = 122.811, *d.f.* = 24,100, *P*<0.01). Based on these results, the order of ingredients in terms of their influence on survival was as follows: honey>sucrose>rapeseed pollen>sinkaline>Tussah pupae>egg yolk. We also confirmed the recipe for optimum survival to be as follows: egg yolk 30 g, Tussah pupa 50 g, sucrose 10 g, honey 15 g, sinkaline 10 g and rapeseed pollen 10 g. We repeated the survival measurements by feeding nymphs with the optimum ADM, which resulted in a 90.7% survival rate.

#### Oviposition duration and female fertility

Both oviposition duration and female life span were significantly affected by the different ingredients (Table 2, *F_1_* = 43.101, d.f. = 24,1078, *P_1_*<0.01; *F_2_* = 42.110, d.f. = 24,1078, *P_2_*<0.01). The oviposition duration across all diets was longer than in the control experiment, whereas female life span was shorter. The order of ingredients in terms of their influence on oviposition duration was as follows: honey>sucrose>rapeseed pollen>egg yolk>sinkaline>Tussah pupa. The optimum ADM combination was: Tussah pupa 50 g, sucrose 10 g, honey 15 g, rapeseed pollen 10 g and with no egg yolk or sinkaline. The oviposition duration of insects fed the optimal ADM was 7.83 d. Egg yolk and honey were the two most important ingredients in terms of improving female fertility. Thus, the order of the remaining ingredients in terms of their effect on fecundity was as follows: rapeseed pollen>Tussah pupa>sucrose>sinkaline. The optimal combination of ingredients, as determined by range analysis, was: egg yolk 40 g, Tussah pupa 50 g, sucrose 10 g, honey 5 g, rapeseed pollen 10 g and no sinkaline. The highest female fertility, 46 new hatched 1^st^ instar larvae, was recorded in *O. sauteri* fed this optimal ADM.

### Bioassay of *O. sauteri* fed by optimal ADMs

#### Respiratory quotient and respiratory rate

**Figure 3:**
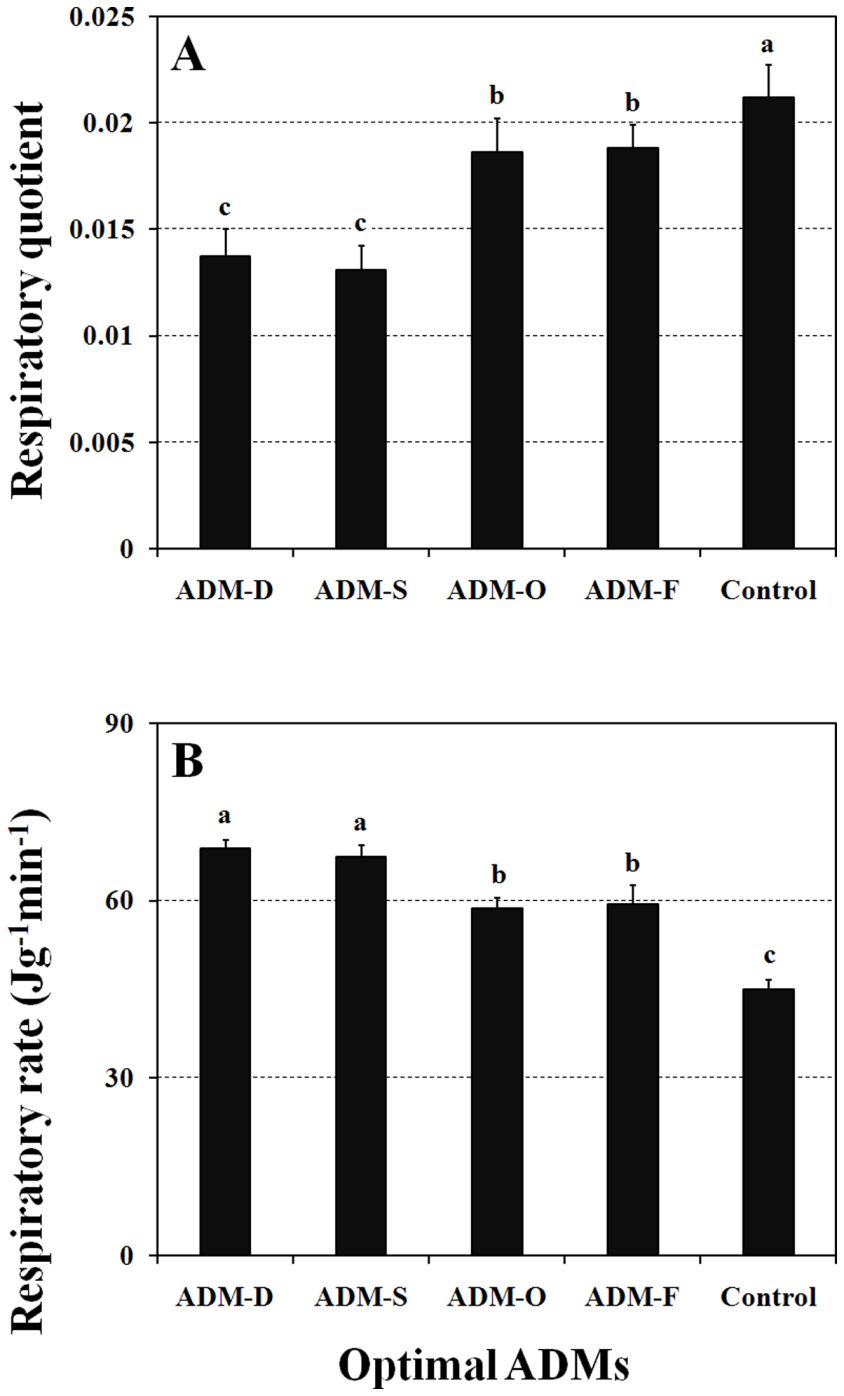
Respiratory quotient and rate of *O. sauteri* fed with either different optimal ADMs or *T. cinnabarinus*. The columns and bars represent mean + SE. The letters at the top of the columns indicate significant differences based on the LSD test (P<0.05).

Once the ingredients of the ADMs had been optimized based on the results of the previous orthogonal experiments, we produced four different types of ADM, which were based on the optimization results of the biological and physiological characters. These ADMs were then fed to all stages from newly hatched female *O. sauteri* nymphs through to adult females. The respiration rate results showed that the average respiratory quotient was significantly different amongst the ADM recipes (Figure 3-A; *F* = 25.477, d.f. = 5, *P*<0.001). The significantly highest respiratory quotient was observed in the control group, whereas the average respiratory quotients of the *O. sauteri* fed on the optimal ADMs for development characteristics (ADM-D and ADM-S) were significantly the lowest. In addition, the two recipes that showed the highest developmental rate also significantly influenced the respiratory rate (Figure 3-B; *F* = 53.912, d.f. = 5, *P*<0.001).

#### Locomotory performance

**Figure 4:**
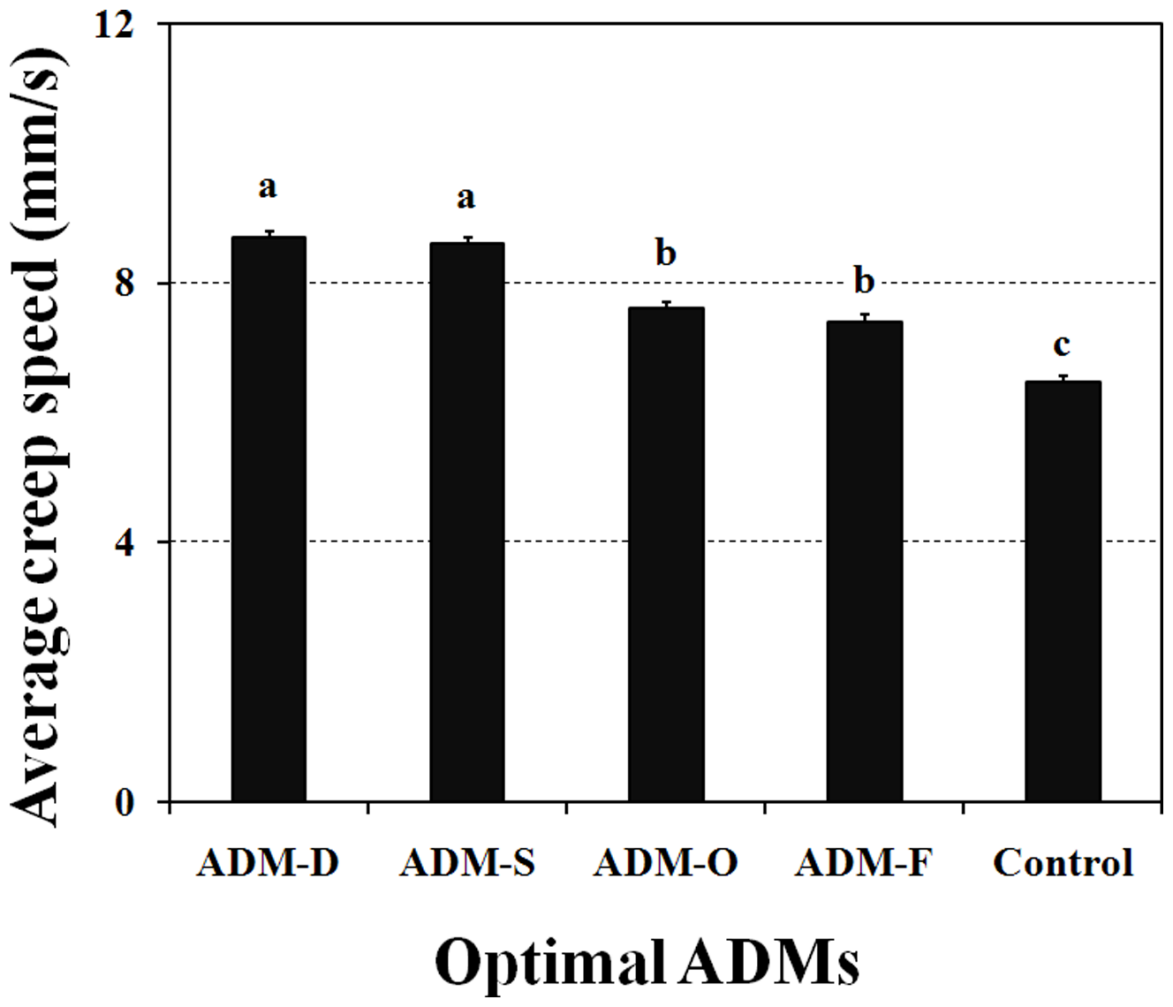
Locomotory performance of *O. sauteri* fed with either different optimal ADMs or *T. cinnabarinus*. The columns and bars represent mean + SE. The different letters at the top of the columns indicate significant differences based on the LSD test (P<0.05).

The results of *O. sauteri* creeping speed variation tracked using the track sphere system are shown in Figure 4. ANOVA showed that ADM type significantly affected the total creep speed (*F* = 64.761, *d.f.* = 5, *P*<0.001). *O. sauteri* fed on the two development-related optimal ADMs showed the highest average creeping speed. The slowest speed was observed with *O. sauteri* fed on *T. cinnabarinus* (i.e. the control group).

#### Mating preference

**Figure 5:**
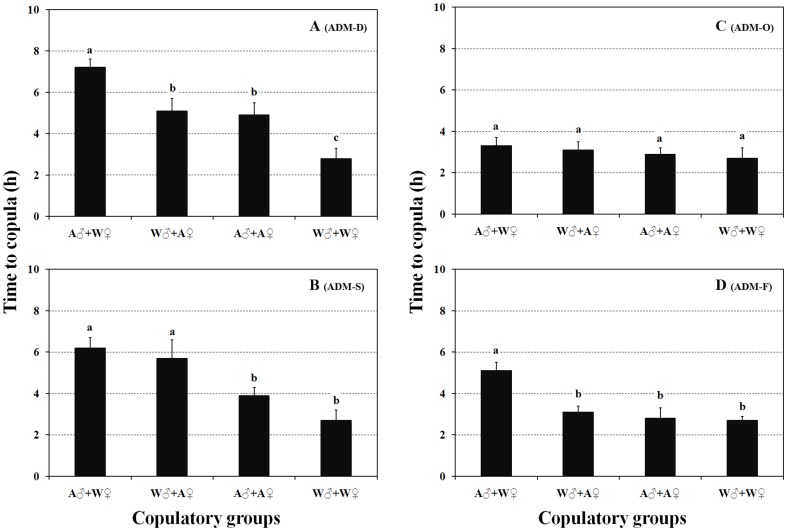
Mating preference (time to copula) of different copulatory groups comprising optimal ADM-reared and wild-collected *O. sauteri* adults (A: artificial reared; W: wild collected). The columns and bars represent mean + SE. The different letters at the top of the columns indicate significant differences based on the LSD test (P<0.05).

The times to copula duration in all four optimal ADMs treatments were significantly longer in ADM ♂ reared+wild ♀ combinations except for the ADM-O treatment (Figure 5 A–D, F_ADM-D_ = 13.427, d.f. = 3,96, P_ADM-D_<0.001; F_ADM-S_ = 7.22, d.f. = 3,94, P_ADM-S_<0.01; F_ADM-O_ = 0.492, d.f. = 3,86, P_ADM-O_ = 0.689; F_ADM-F_ = 9.392, d.f. = 3,92, P_ADM-F_<0.01;). Furthermore in ADM-reared females with a preference for ADM-reared male and wild males, a difference in the time to copula was only found in the optimal ADM-S treatment group.

#### Predatory ability

**Figure 6:**
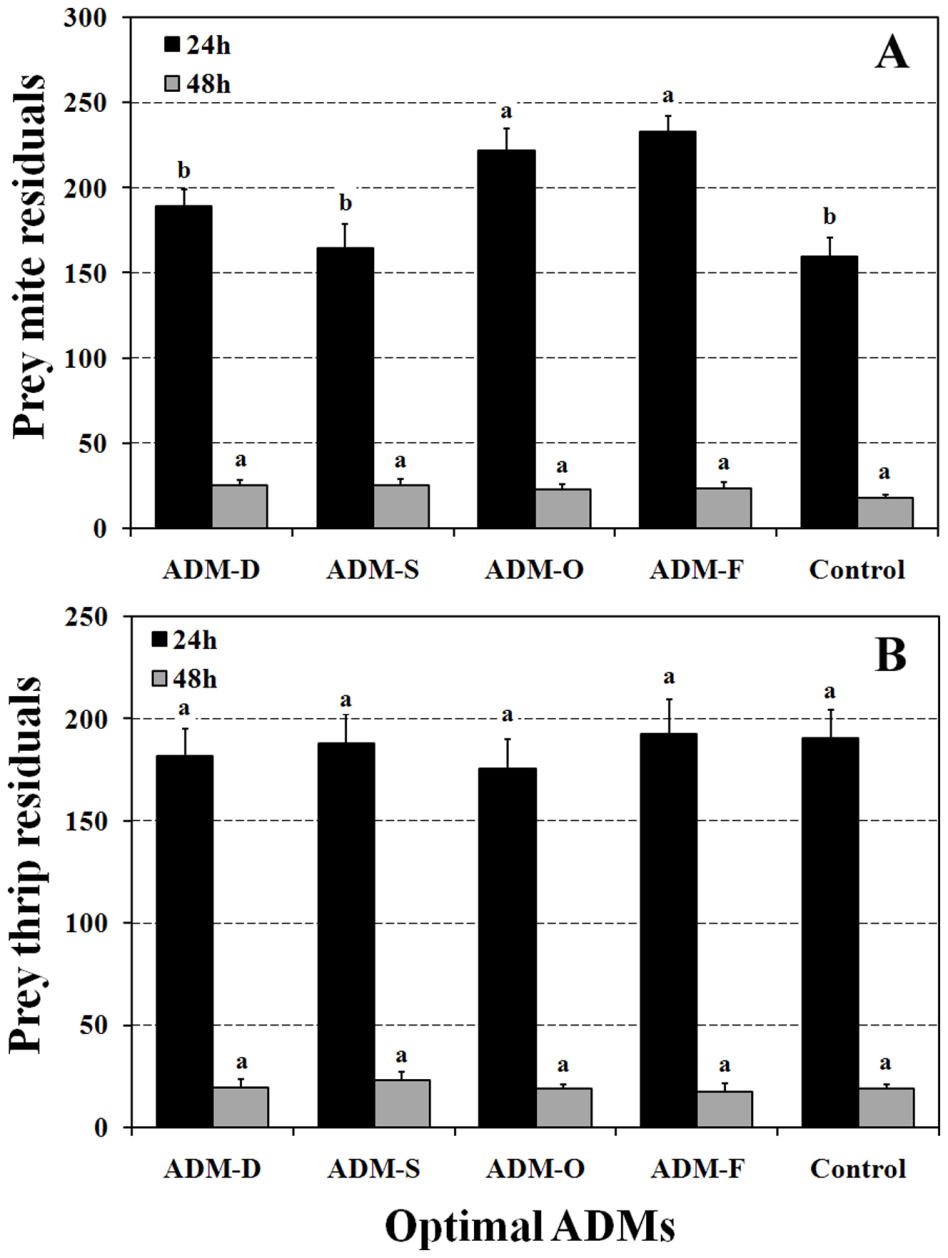
The number of prey leaf mites *T. cinnabarinus* (A) and *F. occidentalis* (B) left after 24 h and 48 h of predation by *O. sauteri* reared on either optimal ADMs or *T. cinnabarinus*. The columns and bars represent mean + SE. The different letters at the top of columns in the same color indicate significant differences based on the LSD test (P<0.05).

The number of remaining prey (*T. cinnabarinus*) was significantly highest in the ADM-O and ADM-F groups than in the other two optimal ADM treatment groups and the control group (Figure 6-A, F = 8.07, d.f. = 4,70, P<0.01). In contrast, optimal ADM treatments did not influence the number of prey remaining compared with the control group (Figure 6-A, F = 1.08, d.f. = 4,70, P = 0.384). The results shown in Figure 6-B indicate that the number of remaining *F. occidentalis* was not influenced significantly by the optimal ADM recipes, either at 24 h (F = 0.227, d.f. = 4,70, P = 0.922) or 48 h (F = 0.354, d.f. = 4,68, P = 0.84).

#### Population dispersal in greenhouses

**Figure 7:**
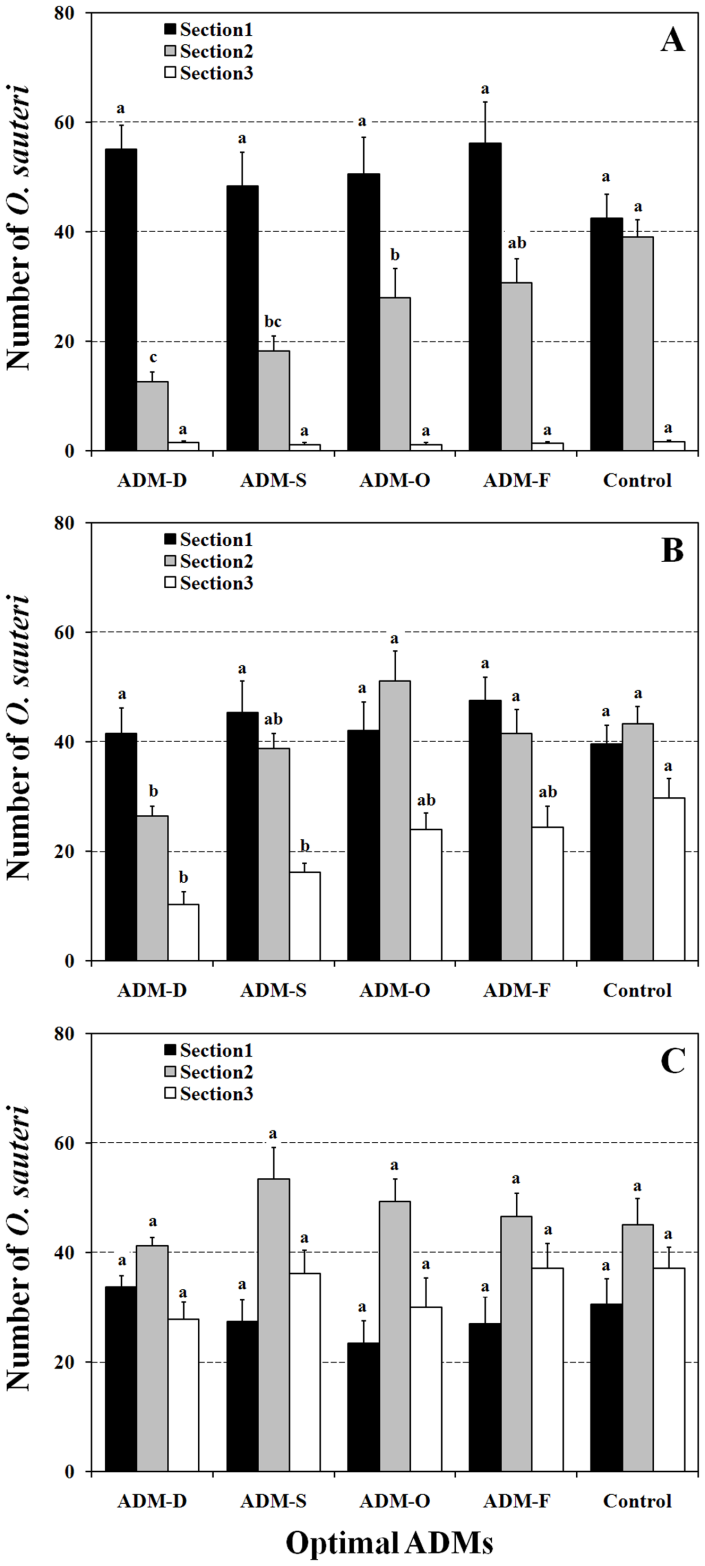
The numbers of *O. sauteri* recorded in the different sections after 24 h (A), 48 h (B) and 72 h (C). The columns and bars represent mean + SE. The different letters at the top of the columns indicate significant differences based on the LSD test (P<0.05).

The number of *O. sauteri* observed on host plants was only significantly different in section 2 at 24 h among all optimal ADM and control groups (Figure 7-A, F_section1_ = 0.843, P_section1_ = 0.505; F_section2_ = 7.908, P_section2_<0.01; F_section3_ = 0.235, P_section3_ = 0.917; all d.f. = 4,45). Compared with the 24-h checkpoint, observations at 48 h showed that the number of *O. sauteri* in sections 2 and 3 were significantly influenced by diet type (Figure 7-B, F_section1_ = 0.443, P_section1_ = 0.771; F_section2_ = 4.23, P_section2_<0.01; F_section3_ = 6.668, P_section3_<0.01; all d.f. = 4,45). Finally, the number of *O. sauteri* did not differ among diet types in all three sections after 72 h (Figure 7-C, F_section1_ = 0.908, P_section1_ = 0.467; F_section2_ = 1.124, P_section2_ = 0.375; F_section3_ = 1.379, P_section3_ = 0.254; all d.f. = 4,45). Further mixed factorial ANOVA highlighted significant interactions of ADM×Section (F = 3.525, d.f. = 8, P<0.01) and Section×Time (F = 55.036, d.f. = 3, P<0.01). However, there was no significant interactive influence of ADMs×Time (F = 1.581, d.f. = 8, P = 1.29) and ADMs×Time×Section (F = 1.751, d.f. = 12, P = 0.065).

## Discussion

The use of an artificial diet to increase the efficiency of mass rearing of *Orius* species has been the focus of study for several decades [39]. Under most experimental conditions, the acceptance of the artificial diet by *Orius* species was the most important determinant of its success [40], [41]. *O. sauteri* have sucking mouthparts and so have more requirements in terms of the form of an artificial diet compared with non-sucking insects [42]. In a previous study, we developed an artificial diet for *O. sauteri* in the form of ADMs and found that such a presentation can have a significant effect on the acceptance of the diet and fitness of the insects [36]. At present, the ADMs eliminated the negative effects of a simple solid or liquid artificial diet on *Orius*, as the microcapsules were found to be more easily accessed by the sucking mouthparts of this genus. Although not presented in this paper, the efficiency of *O. sauteri* on ADMs almost doubled compared with that of insects on a semi-solid artificial diet (data not shown). De Clercq *et al.* [43] demonstrated the stretched Parafilm-made artificial diet that was cylindrical in shape could increase the successful rearing of the spined solider bug *Podisus maculiventris*. Three alternative gelling agents (carrageenan, trehalose and starch) showed a lower level of acceptance by *O. insidiosus*, although the level of thickening achieved was only one-third that of the general gelling agent agarophyte [44]. Previous work demonstrated the AMDs showed advantages in terms of being convenient to store and transport [45]. We were able to conserve the AMD produced for the current study at 10°C for over 110 days, and the appearance of the AMDs in terms of their surface flexibility and glossiness, the quality of the liquid diet they contained, and their shape and size did not show any significant difference. The dome size of the microcapsules in current study was between 0.50 mm and 0.75 mm (average 0.654±0.021 mm, *n* = 4310). The uniform diet dome size is likely to decrease the negative effect of the AMDs on oviposition during mass rearing [46].

We optimized a series of recipes of AMDs for feeding *O. sauteri* according to their effects on various biological and physiological characteristics, by an orthogonal test. Many related studies have revealed that the demands in terms of the diet for the mass rearing of biological control agents can lead to high variation and adjustment of artificial diet recipe components and their proportions [47]. In the current study, all of the ingredients, except the Tussah pupa, were from non-insect sources. Based on our results, we found that the natural organic components (egg yolk and honey) had more positive effects on development and female fertility, although artificial non-insect components had more important effects on nymph survival and oviposition duration. The non-insect ingredients that were added at a high concentration had the most positive effect on most of the test characteristics examined for *O. sauteri*.

Plant farina (cereal grains) can satisfy the nutritional demands of *O. sauteri* throughout its development [39]. However, further research indicated that *O. sauteri* was unable to maintain a stable, high reproductive rate on an artificial diet containing farina as the main ingredient [48]. Pollen was also unable to provide enough nutrition for mass rearing, especially for the reproductive activity of *O. laevigatus* and *O. albidipennis* [20], [49]. *O. insidiosus* was able to develop more successfully with a pollen food supply, although the fecundity of the adults was lower than those fed on arthropod prey [50]. When we investigated the effect of AMD on the reproductive parameters (oviposition duration, and female fertility), we found that decreasing the amount of rapeseed pollen could also have a positive effect on the rearing of *O. sauteri*. There has been a report showing that provision of pollen to *O. laevigatus* under nutrient-limited conditions can avoid cannibalism [51]. However, we did not test the effect of different pollen concentrations on the rate of the cannibalism in *O. sauteri*, although a high cannibalism rate is known to be a feature of this species.

Two studies investigated the addition of sinkaline to artificial diets for the mass rearing of many arthropod species [52], [53]. We observed a more positive effect of sinkaline compared with that of pollen on development, although it had no effect on the rate of reproduction. Other non-insect components, including sucrose and egg yolk, showed normal but necessary effects in every optimal recipe examined, especially on oviposition duration. It also appears from our results that providing saccharides enables the high energy demands of mating to be met successfully. Sucrose is an efficient component in many artificial diets for the mass rearing of arthropods, where it is used as a feeding stimulant [54]. Insect components are necessary for mass rearing of arthropods although the research of insect artificial diet has been carried out for a long period of time [40]. Almost all the artificial diets of *Orius* contained insect tissue or related extracts [27], [28], [44]. Pure artificial diet without any insect components may cause many regression of reared arthropod in development, eclosion, oviposition and fertility [40]. So, we confirmed *A. paphia* pupa as a unique insect component used in the current study, and we found that it had positive effects on all the biological and physiological parameters examined. Lepidopteran eggs and pupae are popular materials for use in the artificial diets for rearing *Orius* species. *Ephestia kuehniella* eggs have been reported to successfully support the development of *O. sauteri* [17] and other *Orius* species [55], [56]. Another lepidopteran, *Plodia interpunctella*, has been used as prey for the direct rearing of *Orius* species [57], [58]. Ferkovich & Shapiro [44] found that introducing an embryo cell line from *P. interpunctella* to the artificial diet could increase the egg-laying rate of *O. insidiosus*. Our results indicate *A. paphia* pupae can improve the development and net fertility of *O. sauteri*. Therefore, future studies should focus on the application of different Lepidopteran resources in artificial diets and investigate the integrated effect of such substances on the mass rearing of arthropods.

The basic requirements for the mass rearing of biological control agents are concerned with not only the quantity of insects produced, but also their quality [59]. Fertility and development have long been seen as key criteria in the mass-rearing industry [27], [28]. Here we used the duration of oviposition and female fertility as additional criteria. The reproduction-specific optimized ADM recipes that had significant positive effects on oviposition duration or net fertility could be used for intensive quality control studies in different stages of *O. sauteri* rearing, and for the evaluation of the effect of different resources. The successful development of biological control needs more efficient methods for artificial mass rearing of insects [32]. The present study focused on dietary factors that had an effect on the biological and physiological characters of *O. sauteri*. This means that the management of key developmental stages during the artificial rearing of *O. sauteri* and other biological control agents is not the only concern for population maintenance, but the functioning of the artificial diet also needs to be considered [60].

We found all biological characters-specific optimized ADMs had positive effects on the metabolic rate of *O. sauteri*, compared with *O. sauteri* fed on leaf mite. The AMDs with significant positive effects on development also resulted in higher respiratory rates than did those that had significant positive effects on oviposition. These results indicate that the differences in nutrition of the AMDs might be responsible for food assimilation. In addition, we observed a significantly higher creeping speed of *O. sauteri* fed on ADMs compared with *O. sauteri* fed on mites. The higher creeping speed in the insects fed ADMs that also had a positive effect on development might result from the developing insects having a higher demand for food compared with the adults that are ovipositing. Our specific ADM is likely to have unexpectedly promoted the correct locomotory capacity.

One of the most important features of successful inoculative or inundative releases of natural enemies during augmentative biological control is the establishment of a stable self-regulating control agent population to ensure a longer period of control management and suppression of the pest population [61]. We showed that, based on reproductive characters, there was no difference in mating performancer between wild females and female *O. sauteri* reared on either of two optimal ADMs; ADM-O and ADM-F. This suggests that some artificial diets can influence the success of population establishment. However, little information is available on the development of control agent populations in the field following their release. Joyce et al. [62] showed that population colonization can influence courtship behaviour in a series of parastoid wasps (Hymenoptera: Braconidae). Jha et al. [63] introduced a life table on the analysis of the potential population development of *Helicoverpa armigera* (Hübner) reared on an artificial diet. The determination of optimal ADMs could help increase the effectiveness of biological control on the basis of different requirements for artificial reproduction or field release [64]. Our results suggest ADM-O and ADM-F could be used for the mass rearing of *Orius* for use in inoculative biological control systems, because of high reproductive and copulatory efficiencies. Although a low efficiency of mating preference of *O. sauteri* reared on ADM-D or ADM-S was recorded, such insects might be more beneficial for inundative augmentative biological control because of a short period of development, that could permit simultaneous mass release.

We did not observe significant differences in prey consumption in *T. cinnabarinus* and *F. occidentalis* among groups fed either optimal ADMs or a natural prey diet (Figure 6-B) at either 24 h or 48 h. Significant differences in predation levels were observed for ADM-O- and ADM-F-fed *O. sauteri* at 24 h, but there was no difference at 48 h. These results suggest that in terms of reproduction, optimal ADMs could provide sufficient benefits in practical field application against various target pests. More remarkably, in terms of development, associated optimal ADMs fed *O. sauteri* could have a role in the efficient suppression of pests in the field. Our observations also suggest that the difference in prey consumption among diets evens out over time. Furthermore, the results of dispersal observation also demonstrated the suitability of ADM rearing for use in practical augmentative biological control. Initially, *O. sauteri* showed a short period of dispersal in association with being fed ADMs. However, after 72 h, there were no differences in population dispersal among the different diet groups. Thus, dispersion of *O. sauteri* might be correlated with variation in prey density. Our results indicate that introduction of ADM-reared *O. sauteri* in the field would require an initial short period of stabilization. In addition, two reproduction-related ADMs would be more beneficial to the preparation of populations for field release. Practical evaluation of these mass-reared natural enemies needs to be promoted in current biological systems [64], although many studies are available that have been performed under laboratory conditions [65]–[66].

## Conclusion

The use of a microencapsulated artificial diet overcame many of the negative effects recorded by previous studies on the use of artificial diets for the mass rearing of *O. sauteri* in terms of having a uniform shape, liquid diet stabilization and so on. The different ADM recipes optimized for specific biological characters were able to directly increase those corresponding biological and physiological characters themselves. The specifically optimized ADMs could enable *O. sauteri* to be reared in a customized culture set-up. The introduction of sinkaline and honey was beneficial in terms of their effects on the biological and physiological characters examined, compared with traditional artificial diet recipes. We also found that an alternative lepidopteran diet ingredient, pupae of *A. paphia*, had beneficial effects on the mass rearing of *O. sauteri*. Intensive respiratory and locomotory tests were used to evaluate the end effect of the different artificial diet recipes. Successive examinations of mating preference, predatory ability and population dispersion showed great benefits for practical biological control application by using the present optimal ADMs. However, more studies are required to further determine the efficacies of such artificial diets in the mass rearing of *O. sauteri* and other insects for biological control.
